# LncRNA ST6GALNAC3 inhibits dermal fibroblast proliferation and migration in cashmere goat hair follicles via the chi-miR-24-3p/ID4 axis

**DOI:** 10.5713/ab.25.0115

**Published:** 2025-06-24

**Authors:** Rong Ma, Qing Ma, Lu Zhang, Bingjie Ma, Xuxu Bao, Yiming Zhang, Le Wang, Qi Lv, Zhiying Wang, Ruijun Wang, Rui Su, Yanhong Zhao, Fangzheng Shang, Yu Wang, Yanjun Zhang

**Affiliations:** 1 College of Animal Science, Inner Mongolia Agricultural University, Hohhot, China; 2 Science and Technology Development Center of Ulanqab, Ulanqab, China; 3 College of Veterinary Medicine, Inner Mongolia Agricultural University, Hohhot, China; 4 Key Laboratory of Mutton Sheep Genetics and Breeding, Ministry of Agriculture, Hohhot, China; 5 Key Laboratory of Goat and Sheep Genetics, Breeding and Reproduction in Inner Mongolia Autonomous Region, Hohhot, China

**Keywords:** Cashmere Goats, Dermal Fibroblast, Hair Follicle, LncRNA, Morphogenesis

## Abstract

**Objective:**

Dermal papilla is developed from the continuous proliferation and differentiation of dermal fibroblasts, which is the key to the normal development of hair follicles. This study aims to elucidate the role of lncRNA ST6GALNAC3, which is significantly differentially expressed during the secondary hair follicle development stage in cashmere goats, on dermal fibroblasts, and to analyze the regulatory mechanism of this lncRNA thoroughly.

**Methods:**

We conducted a screening process and characterization for lncRNAs associated with the development of secondary hair follicles. The effects of lncRNA ST6GALNAC3 on cell proliferation and migration were assessed using CCK8, EdU, and flow cytometry. Subsequently, we employed bioinformatics analysis to identify the target miRNAs of lncRNA ST6GALNAC3 and the corresponding target genes of these miRNAs, respectively, and initially constructed the regulatory axis of lncRNA ST6GALNAC3-chi-miR-24-3p-ID4. Luciferase reporter assays and rescue experiments were performed to confirm the regulatory axis at both molecular and cellular levels, thus elucidating the mechanism by which lncRNA ST6GALNAC3 regulates dermal fibroblasts.

**Results:**

One hundred fifty-eight lncRNAs related to secondary hair follicle morphogenesis were identified. Among them, lncRNA ST6GALNAC3 was significantly differentially expressed on embryonic day 75 and significantly inhibited the proliferation and migration of dermal fibroblasts. The results showed that lncRNA ST6GALNAC3 can target chi-miR-24-3p, which in turn can target the *ID4* gene. The results of the luciferase reporter assay and rescue assay showed that chi-miR-24-3p binds to both lncRNA ST6GALNAC3 and *ID4*. Furthermore, lncRNA ST6GALNAC3 can indirectly regulate the proliferation and migration of dermal fibroblasts through chi-miR-24-3p/ID4 axis.

**Conclusion:**

LncRNA ST6GALNAC3 inhibits the proliferation and migration of dermal fibroblasts through the chi-miR-24-3p/ID4 axis, thus suppressing the formation of dermal papilla structures and influencing the morphogenesis of secondary hair follicles during embryonic development.

## INTRODUCTION

Cashmere goats are a distinctive livestock species with significant importance in the global animal husbandry sector. They are distributed within the region between 25°–55° north latitude and 40°–125° east longitude, including countries such as China, Mongolia, Kyrgyzstan, Iran, and India [1]. In China, they are mainly found in the southwest, northwest, north, and northeast regions, with the five major pastoral areas of Inner Mongolia, Xinjiang, Qinghai, Gansu, and Tibet being the primary locations. Inner Mongolia cashmere goats are an excellent local breed primarily bred. The cashmere they produce is fine and soft, making it an extremely precious natural fiber [2]. Textiles made from cashmere are lightweight, soft, and highly effective at retaining heat, making them highly sought-after by consumers. Cashmere grows from the secondary hair follicles of cashmere goats, and its production is relatively low. According to statistics, the annual global production of cashmere only accounts for about 0.2% of the total animal fiber production, which keeps the market price of cashmere consistently high. High-quality cashmere products are even more expensive, becoming an important part of the luxury market. This has also brought considerable economic benefits to farmers, with many regions achieving poverty eradication and prosperity through the cashmere goat breeding industry, thereby promoting local economic development.

The hair follicle is a unique skin appendage in mammals, characterized by its relatively complex morphology, and it plays a significant role in maintaining skin homeostasis, thermoregulation, and metabolism [3]. Additionally, they regulate hair growth and determine the quality and yield of hair, both of which are crucial economic traits for animals that produce cashmere. The formation of hair follicles is particularly critical during embryogenesis, as the development at this stage will directly influence the quality and yield of cashmere [4]. Therefore, a thorough exploration of the developmental mechanisms of cashmere goat hair follicles is of great significance for improving cashmere quality, increasing production, and even promoting the prosperity and development of the entire cashmere industry. Cashmere goats’ skin contains primary and secondary hair follicles [5]. Secondary hair follicles are the key organs for growing cashmere. Studies have shown that during the development of cashmere goat hair follicles, primary hair follicles always precede secondary hair follicles [6]. Starting from the 45th day of embryogenesis, the fetal skin has initially formed a complete epidermal structure. By the 55th day of embryogenesis, primary hair follicle bud structures begin to appear. As development continues, by the 65th day, these hair follicle buds continue to grow downward. By the 75th day, secondary hair follicle bud structures emerge for the first time [7,8].

The development of hair follicles is a complex physiological process that relies on intricate signaling between the dermis and epidermis to form a complete hair follicle structure [3]. Among them, the dermal papilla, derived from the proliferation and differentiation of dermal fibroblasts, serves as the signaling hub within the entire hair follicle structure and is crucial for ensuring normal hair follicle growth [9]. In recent years, with the rapid development of biotechnology and the increasing popularity of high-throughput sequencing technology, more and more complex biological processes have been explored in depth, and numerous important signaling molecules related to hair follicle development have been discovered. Long non-coding RNAs (lncRNAs) were initially considered by many researchers to be noise in RNA transcription processes [10]. However, with continuous innovations in biological technology, researchers have found that lncRNAs can affect mRNA expression directly or indirectly, thereby regulating processes such as hair follicle development and hair growth. For example, studies have found that lncRNA MSTRG.20890.1 and lncRNA MSTRG.14227.1 can inhibit the expression of the *ADAMTS3* gene by targeting and binding to miRNAs, thus inhibiting the proliferation and migration of dermal fibroblasts in cashmere goats [1,6]. LncRNA FABP_AS competitively binds with chi-miR-335-5p to promote *DKK1* gene expression, reduce Wnt/β-catenin signaling pathway activity, and thereby inhibit the proliferation of hair follicle stem cells [11]. Zhang et al identified a total of 521 differentially expressed lncRNAs in cashmere goats at different embryonic stages using high-throughput sequencing technology. Among them, lncRNA H19 can significantly promote the proliferation of dermal papilla cells, and this promotional effect is regulated through the chi-miR-214-3p/β-catenin axis [12]. Currently, many signaling molecules related to hair follicle development in cashmere goats have been gradually identified by researchers. However, studies further exploring lncRNAs related to secondary hair follicle development and their regulatory mechanisms are still scarce. Therefore, based on the skin transcriptome database of cashmere goats at different embryonic stages previously constructed by our research group [13], we screened 158 lncRNAs related to secondary hair follicle morphogenesis. Through identification and analysis, we found that lncRNA ST6GALNAC3 was significantly under-expressed at the onset of secondary hair follicle development (embryonic day 75). This lncRNA upregulates the expression of the *ID4* gene by sponging chi-miR-24-3p, thereby inhibiting the proliferation and migration of dermal fibroblasts. In addition, we found that this inhibitory effect on cell proliferation was achieved by decreasing the proportion of cells in the S phase. In summary, our study demonstrates that lncRNA ST6GALNAC3 promotes the expression of *ID4* in dermal fibroblasts by binding to chi-miR-24-3p, thereby regulating the morphogenesis of secondary hair follicles in cashmere goats. This study further reveals the regulatory mechanism of lncRNAs in the development of secondary hair follicles in cashmere goats and provides a theoretical basis for improving the quality and yield of cashmere in cashmere goats.

## MATERIALS AND METHODS

### Skin sample collection

Experimental animals were obtained from the Inner Mongolia Jinlai Animal Husbandry Technology (Hohhot, China). Skin samples were obtained from cashmere goat embryos at 45, 55, 65, and 75 days. These samples were stored at −80°C.

### Screening of lncRNAs related to secondary hair follicle morphogenesis

Based on the previously constructed skin transcriptome database of cashmere goats at different embryonic stages (45 days, 55 days, 65 days, and 75 days) [13], we analyzed the differentially expressed lncRNAs across these stages using the criteria of |log2foldchange|≥1 and p-value≤0.05 as selection thresholds. Previous research by our team has revealed that, at 45 days of embryonic development, the skin of cashmere goats possesses only a complete epidermal structure, lacking any hair follicle structures. Primary hair follicles initiate their development at 55 days of embryonic development and continue to extend towards the dermal layer as the fetus develops, ultimately resulting in the formation of a complete primary hair follicle structure before birth. The development of secondary hair follicles lags behind that of primary hair follicles, initiating their formation at 75 days of embryonic development and continuing to develop until a complete secondary hair follicle structure is formed before birth [7,8]. According to the characteristics of hair follicle development described above, embryonic day 55 was considered to be the key point for primary hair follicle morphogenesis, and embryonic day 75 was considered to be the key point for secondary hair follicle morphogenesis. We classified three comparison groups (d55 vs d45, d65 vs d45, and d65 vs d55) as Stage 1, which is associated with the growth and development of primary hair follicles. The other three comparison groups (d75 vs d45, d75 vs d55, and d75 vs d65) were classified as Stage 2, which is related to both primary and secondary hair follicle development. Subsequently, we took the intersection of Stage 1 (primary hair follicles) and Stage 2 (primary hair follicles+ secondary hair follicles) and excluded this intersection from Stage 2. The remaining lncRNAs in Stage 2 were considered important lncRNAs related to the morphogenesis of secondary hair follicles.

### Cell culture and transfection

Dermal fibroblasts were cultured in a medium containing 10% fetal bovine serum. The culture conditions for all cell lines were maintained at 37°C with 5% CO_2_. Lentiviral transfection was employed to deliver the target plasmid into dermal fibroblasts, and the target plasmid was prepared by Shanghai Hanheng Biotechnology (Shanghai, China). After transfection, puromycin was added to perform antibiotic selection.

### Subcellular localization

LncLocator [14] predicted lncRNA ST6GALNAC3’s location. Cytoplasmic and nuclear RNA from dermal fibroblasts were extracted using the Cytoplasmic & Nuclear RNA Purification Kit (Norgen Biotek, Thorold, ON, Canada), followed by quantitative realtime reverse transcription polymerase chain reaction (qRT-PCR) to detect lncRNA ST6GALNAC3 expression in these locations.

### FISH assay

Prepare the cells to the appropriate density. The cells were then treated with 4% paraformaldehyde for 20 min, prehybridization solution for 1 h, and then incubated with a probe mix overnight. Subsequently, the prehybridization solution was washed off, and blocking serum (BSA) was added. The cells were incubated with the BSA for 30 min at room temperature. Finally, the cells were incubated with DAPI staining solution for 8 min, washed to remove the staining solution, and observed under a fluorescence microscope.

### Prediction of targeting relationships

Sequence information of lncRNA and mRNA was obtained using the previously constructed cashmere goat skin transcriptome database. The sequence information of miRNA was obtained from the miRBase database. In this study, miRanda [15] and TargetScan [16] software were used to predict the target miRNAs of lncRNA and target genes of miRNA, respectively. miRanda [15] is mainly based on the free binding energy between lncRNA and miRNA, while TargetScan [16] is primarily based on seed sequence to predict the targeting relationship between mRNA and miRNA. The sequence information of lncRNA and miRNA was input into the miRanda [15] software. When the threshold was less than −10, it indicated a targeted binding relationship between them (the smaller the threshold, the greater the possibility of interaction between them). The sequence information of mRNA and miRNA was input into the TargetScan [16] software. When the TargetScan threshold exceeded 50, it was inferred that there might be a targeted binding relationship between them (the higher the threshold, the greater the likelihood of interaction between them). Subsequently, the RNAhybrid software (v2.1.2) was employed to validate the lncRNA-miRNA and miRNA-mRNA targeting relationships identified earlier. When the binding free energy of a targeting relationship pair was less than −20 kcal/mol, we preliminarily believed that there was a strong binding ability between them (the lower the free binding energy, the stronger the binding ability).

### Dual-luciferase report detection

The dual-luciferase reporter system was used to verify the targeting relationship in 293T cells. Cells were co-transfected with chi-miR-24-3p mimic and either psiCHECK2-lncRNA ST6GALNAC3-WT/MUT or psiCHECK2-ID4-WT/MUT using LipoFiter (Hanheng, Shanghai, China). Luciferase activity was measured 48 h post-transfection (Promega, Madison, WI, USA).

### Cell proliferation assays

Cell proliferation was assessed by CCK8 and EdU methods. CCK8 measured proliferation using a kit (Solarbio, Beijing, China), with OD read at 450 nm. The EdU assay was performed based on the instructions of the BeyoClick EdU-555 Cell Proliferation Detection Kit (Beyotime, Shanghai, China). The EdU assay calculates proliferation as the ratio of EdU-labeled cells to Hoechst-labeled cells.

### Cell apoptosis detection

Cell apoptosis was determined using the Annexin V-APC/PI Kit (Elabscience Biotechnology, Wuhan, China). A single-cell suspension was prepared and stained with reagents. After incubating in the dark for 20 min, cell apoptosis was detected by flow cytometry.

### DNA staining

The cell cycle was determined by the DNA content quantification method (Solarbio). Cells were fixed in 70% ethanol, resuspended, and incubated at 4°C. After 24 h, RNase A was added, and cells were incubated at 37°C for 30 min. The PI staining solution was then added, and cells were incubated at 4°C in the dark for 30 min.

### Cell scratch assay

The cell scratch assay involved scraping a cell monolayer and capturing images at 0 h and 24 h post-scratch. Cells were washed with PBS, added fresh serum-free medium, and incubated. Cell migration rate was calculated as ([0 h area–24 h area]/0 h area)×100%.

### Statistical analysis

Statistical analysis was conducted using SPSS 26.0 (IBM, Armonk, NY, USA) and GraphPad Prism 8.0 (GraphPad Software, San Diego, CA, USA). All data are presented as mean±SD from ≥3 independent experiments. Significance was determined by Student’s t-tests with p<0.05 or p<0.01.

## RESULTS

### Screening for lncRNAs related to the morphogenesis of secondary hair follicles in cashmere goats

**Figure 1:**
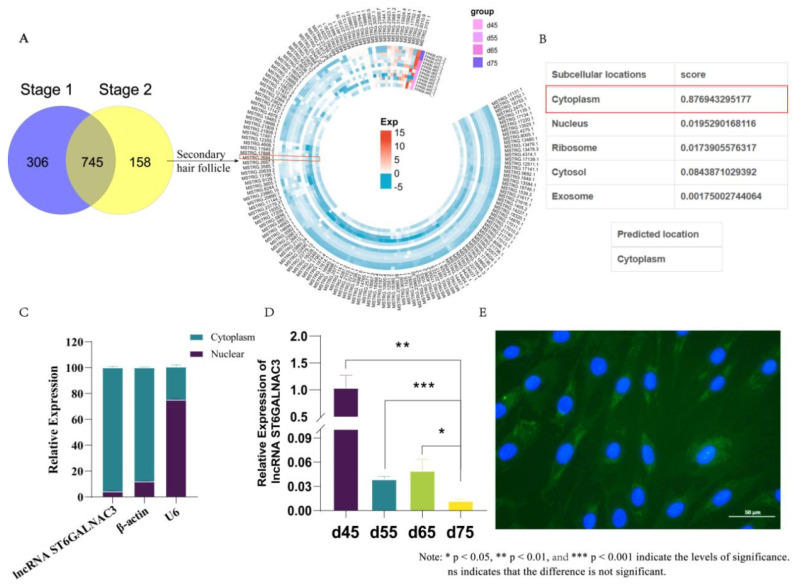
Screening and identification of lncRNAs related to secondary hair follicle morphogenesis. (A) Screening of lncRNAs associated with secondary hair follicle morphogenesis. (B) lncLocator software predicts the distribution of lncRNA ST6GALNAC3 in cells. (C) Detection of lncRNA ST6GALNAC3 expression in the nucleus and cytoplasm of dermal fibroblasts. (D) Expression of lncRNA ST6GALNAC3 in skin tissues at different embryonic periods. (E) The distribution of lncRNA ST6GALNAC3 in cells was detected by FISH assay.

To identify lncRNAs related to the morphogenesis of secondary hair follicles in cashmere goats, we screened and analyzed the transcriptome database of skin tissues from different embryonic stages that had been previously established [13]. Initially, we analyzed the differentially expressed lncRNAs across these stages using the criteria of p-value≤0.05 and |log2foldchange|≥1. Through differential comparison analysis, we identified a total of 1,209 lncRNAs that were differentially expressed in skin tissues at different embryonic stages. Previous research by our team has revealed that, at 45 days of embryonic development, the skin of cashmere goats possesses only a complete epidermal structure, lacking any hair follicle structures. Primary hair follicles initiate their development at 55 days of embryonic development and continue to extend towards the dermal layer as the fetus develops, ultimately resulting in the formation of a complete primary hair follicle structure before birth. The development of secondary hair follicles lags behind that of primary hair follicles, initiating their formation at 75 days of embryonic development and continuing to develop until a complete secondary hair follicle structure is formed before birth [7,8]. The morphogenesis of secondary hair follicles determines the number of secondary hair follicles in cashmere goats, which subsequently influences the production of cashmere [5]. Therefore, based on the characteristics of hair follicle morphological development in the embryonic period, we considered embryonic day 55 as the key time node for primary hair follicle morphogenesis and embryonic day 75 for secondary hair follicle morphogenesis. We designated three comparison groups (d55 vs d45, d65 vs d45, d65 vs d55) as Stage 1, which encompassed a total of 1,051 lncRNAs associated with the development of primary hair follicles. Additionally, we designated the other three comparison groups (d75 vs d45, d75 vs d55, d75 vs d65) as Stage 2, which encompassed a total of 903 lncRNAs related to the morphogenesis of both primary and secondary hair follicles. To identify lncRNAs specifically associated with secondary hair follicle morphogenesis, we first determined the intersection of lncRNAs present in both Stage 1 and Stage 2. These 745 overlapping lncRNAs are likely involved in processes common to both primary and secondary hair follicle development. By excluding these 745 lncRNAs from the 903 lncRNAs in Stage 2, we obtained a set of 158 lncRNAs that were considered important for the morphogenesis of secondary hair follicles (Figure 1A). Among these 158 lncRNAs, we discovered that lncRNA ST6GALNAC3 was highly expressed in samples at 45, 55, and 65 days of the embryonic period, while it was down-regulated at 75 days of the embryonic period. This lncRNA exhibits good biological reproducibility. The 75th day of the embryonic stage is a crucial period for the morphogenesis of secondary hair follicles in cashmere goats. Therefore, we further examined the expression of lncRNA ST6GALNAC3 in skin tissues at different periods using qRT-PCR. Consistent with the sequencing results, the expression of lncRNA ST6GALNAC3 was significantly lower at embryonic day 75 compared with other stages (Figure 1D). Thus, we speculate that this lncRNA may be related to the morphogenesis of secondary hair follicles.

Simultaneously, we further examined the coding potential and cellular distribution of lncRNA ST6GALNAC3. To analyze the coding ability of lncRNA ST6GALNAC3, we utilized CPC [17] and CNCI [18] software. The results showed CPC [17] and CNCI [18] scores of 0.1 and −0.02, respectively, indicating that lncRNA ST6GALNAC3 lacks protein-coding capacity. LncLocator [14] software predicted that lncRNA ST6GALNAC3 is primarily located in the cytoplasm (Figure 1B). Subsequently, we utilized FISH and nuclear-cytoplasmic separation experiments to detect the distribution of lncRNA ST6GALNAC3 in dermal fibroblasts. The experimental results were consistent with the predictions, revealing that this lncRNA is mainly localized in the cytoplasm of dermal fibroblasts (Figures 1C, 1E).

### Suppressing the expression of lncRNA ST6GALNAC3 can enhance the proliferation and migration of dermal fibroblasts

**Figure 2:**
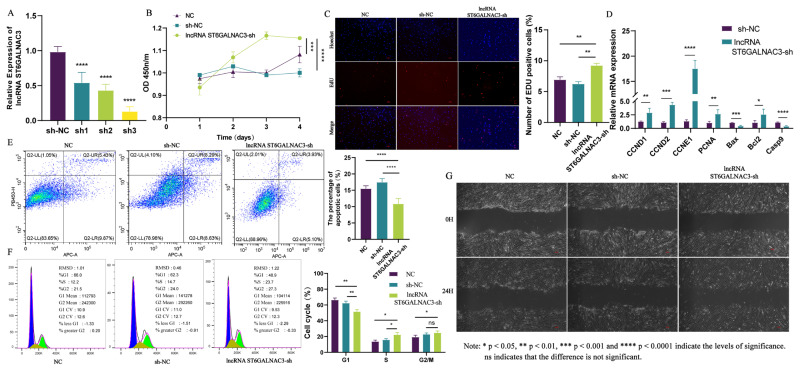
Functional analysis of lncRNA ST6GALNAC3 in dermal fibroblasts. (A) Screening of lncRNA ST6GALNAC3 interference vector. (B) CCK8 was used to detect the proliferation of the lncRNA ST6GALNAC3-sh cell line. (C) The EdU was used to detect the proliferation of the lncRNA ST6GALNAC3-sh cell line. (D) The expression of cell proliferation/apoptosis marker genes was detected by qRT-PCR. (E) The apoptosis of dermal fibroblasts was detected after lncRNA ST6GALNAC3 interference. (F) DNA staining was used to detect the cell cycle of the lncRNA ST6GALNAC3-sh cell line. (G) The cell scratch assay was used to detect the migration of the lncRNA ST6GALNAC3-sh cell line. qRT-PCR, quantitative real-time reverse transcription polymerase chain reaction.

The morphogenesis and development of hair follicles result from the continuous proliferation and differentiation of dermal fibroblasts and epithelial cells. Among them, dermal fibroblasts ultimately differentiate to form the dermal papilla structure, which serves as the signaling hub for hair follicle growth and regulates the development of the entire hair follicle. Therefore, we utilized lentiviral transfection to introduce lncRNA ST6GALNAC3-sh1/2/3 vectors into dermal fibroblasts and subsequently evaluated their interference efficiency. We found that the lncRNA ST6GALNAC3-sh3 vector exhibited the best interference efficiency, and thus selected it for subsequent cellular phenotypic experiments (Figure 2A).

Firstly, we utilized EdU assays and CCK8 methods to examine the impact of lncRNA ST6GALNAC3 interference on the proliferation of dermal fibroblasts. The results of the cell proliferation experiments indicated that lncRNA ST6GALNAC3-sh could increase the proportion of EdU-positive cells in dermal fibroblasts, and the proliferative capacity of these cells was also significantly enhanced compared to that of the control group (Figures 2B, 2C). Subsequently, flow cytometry was used to detect changes in the cell cycle and apoptosis, further investigating the role of lncRNA ST6GALNAC3 in promoting cell proliferation. The experimental results indicated that the knockdown of lncRNA ST6GALNAC3 significantly decreased the apoptotic rate of dermal fibroblasts while significantly increasing the proportion of cells in the S phase of the cell cycle (Figures 2E, 2F). Additionally, we further examined the expression of genes related to proliferation and apoptosis following lncRNA ST6GALNAC3 knockdown. qRT-PCR results demonstrated that the lncRNA ST6GALNAC3-sh could upregulate the expression of *CCND1*, *CCNE1*, *CCND2*, and *PCNA* (proliferation-related genes), while downregulating the expression of *Bax* and *Casp9* (apoptosis-related genes) (Figure 2D). In summary, lncRNA ST6GALNAC3-sh can promote the proliferation of dermal fibroblasts, and this promotional effect may be mediated by increasing the proportion of cells in the S phase and enhancing the expression of cell proliferation-related genes. Dermal fibroblasts not only continuously proliferate and differentiate but also migrate further into the dermis of the skin, ultimately forming the dermal papilla structure at the base of the hair follicle. Therefore, this study utilized a cell scratch assay to further investigate the impact of lncRNA ST6GALNAC3 on cellular migration ability (Figure 2G). The results showed that the migration ability of dermal fibroblasts was significantly increased following transfection with the lncRNA ST6GALNAC3-sh. In conclusion, the lncRNA ST6GALNAC3-sh enhances the proliferation and migration ability of dermal fibroblasts, thereby regulating the formation of the dermal papilla.

### LncRNA ST6GALNAC3 enhance *ID4* expression via absorbing chi-miR-24-3p

**Figure 3:**
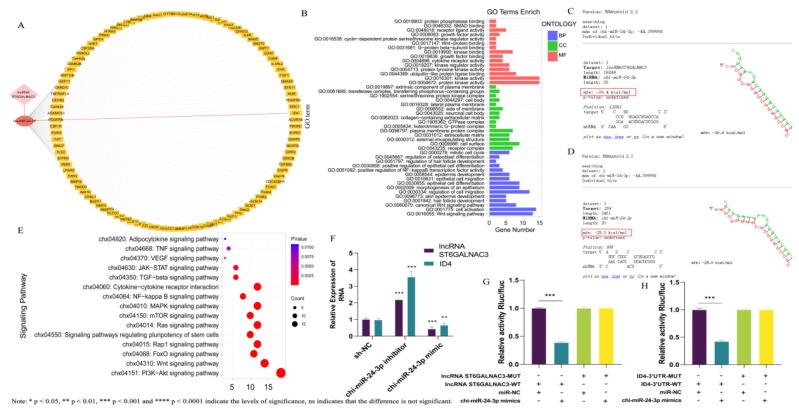
Construction and verification of lncRNA ST6GALNAC3-chi-miR-24-3p-ID4 regulatory network. (A) Construction of a regulatory network. (B) GO enrichment analysis of chi-miR-24-3p target genes. (C) RNAhybrid software predicts the sequence of lncRNA ST6GALNAC3 binding site to chi-miR-24-3p. (D) RNAhybrid software predicts the sequence of the *ID4* binding site to chi-miR-24-3p. (E) KEGG enrichment analysis of chi-miR-24-3p target genes. (F) chi-miR-24-3p interference/overexpression of dermal fibroblast cell lines to detect lncRNA ST6GALNAC3/ID4 expression. (G) Dual-luciferase reporter gene system to detect target binding of lncRNA ST6GALNAC3 to chi-miR-24-3p. (H) Dual-luciferase reporter gene system to detect target binding of *ID4* to chi-miR-24-3p.

Numerous studies on lncRNAs have demonstrated that their intracellular localization is closely associated with their regulatory functions. Typically, lncRNAs located in the cytoplasm often indirectly regulate the expression of related genes by sponging miRNAs. Preliminary experimental results suggest that lncRNA ST6GALNAC3 is mainly located in the cytoplasm of cells. Consequently, we hypothesize that lncRNA ST6GALNAC3 may act as a ceRNA to regulate the expression of related genes. Firstly, we conducted bioinformatics analysis utilizing miRanda [15] and found potential binding sites between the lncRNA ST6GALNAC3 and chi-miR-24-3p. Furthermore, by employing the RNAhybrid software (v2.1.2), we acquired sequence information about the binding sites between lncRNA ST6GALNAC3 and chi-miR-24-3p. The binding free energy between the two entities was computed to be lower than −20 kcal/mol, which signifies a robust binding capacity (Figure 3C). At the same time, we predicted the target genes of chi-miR-24-3p and found that it has a potential binding capacity with 103 mRNAs. Figure 3A displays their regulatory network. Next, we did enrichment analysis on chi-miR-24-3p’s target genes (Figures 3B, 3E). The results showed these genes were mainly in pathways related to hair follicle development, like TGF-β, Wnt, and PI3K-AKT. This further indicates that lncRNA ST6GALNAC3 can act as a ceRNA to regulate the expression of genes related to hair follicle development, and then indirectly participate in the process of hair follicle development. Among these target genes, we selected the *ID4* gene, which is enriched in the TGF-β signaling pathway, for subsequent experimental validation and further analysis. The prediction results from RNAhybrid (v2.1.2) indicate that the binding free energy between *ID4* and chi-miR-24-3p is −28 kcal/mol, which is less than −20 kcal/mol, suggesting a strong binding capacity between them (Figure 3D). Subsequently, we employed qRT-PCR to examine the expression levels of lncRNA ST6GALNAC3 and *ID4* in cell lines with chi-miR-24-3p knockdown/overexpression. The experimental results showed that both lncRNA ST6GALNAC3 and *ID4* were highly expressed in the chi-miR-24-3p knockdown cell lines, whereas they were lowly expressed in the chi-miR-24-3p overexpression cell lines (Figure 3F). This phenomenon is consistent with the ceRNA hypothesis. Furthermore, we constructed wild-type and mutant dual-luciferase reporter plasmids for lncRNA ST6GALNAC3 and ID4-3’UTR, respectively, and employed the dual-luciferase reporter gene system to detect the targeting relationships between lncRNA ST6GALNAC3 and chi-miR-24-3p, as well as between the ID4-3’UTR and chi-miR-24-3p. The results showed that the chi-miR-24-3p mimic significantly decreased the luciferase activity of both the wild-type lncRNA ST6GALNAC3 reporter gene and the wild-type ID4-3’UTR reporter gene, whereas there was no significant change in the mutant versions, indicating that both lncRNA ST6GALNAC3 and *ID4* can bind to chi-miR-24-3p (Figures 3G, 3H).

### Inhibition of *ID4* expression can promote the prolife-ration and migration of dermal fibroblasts

**Figure 4:**
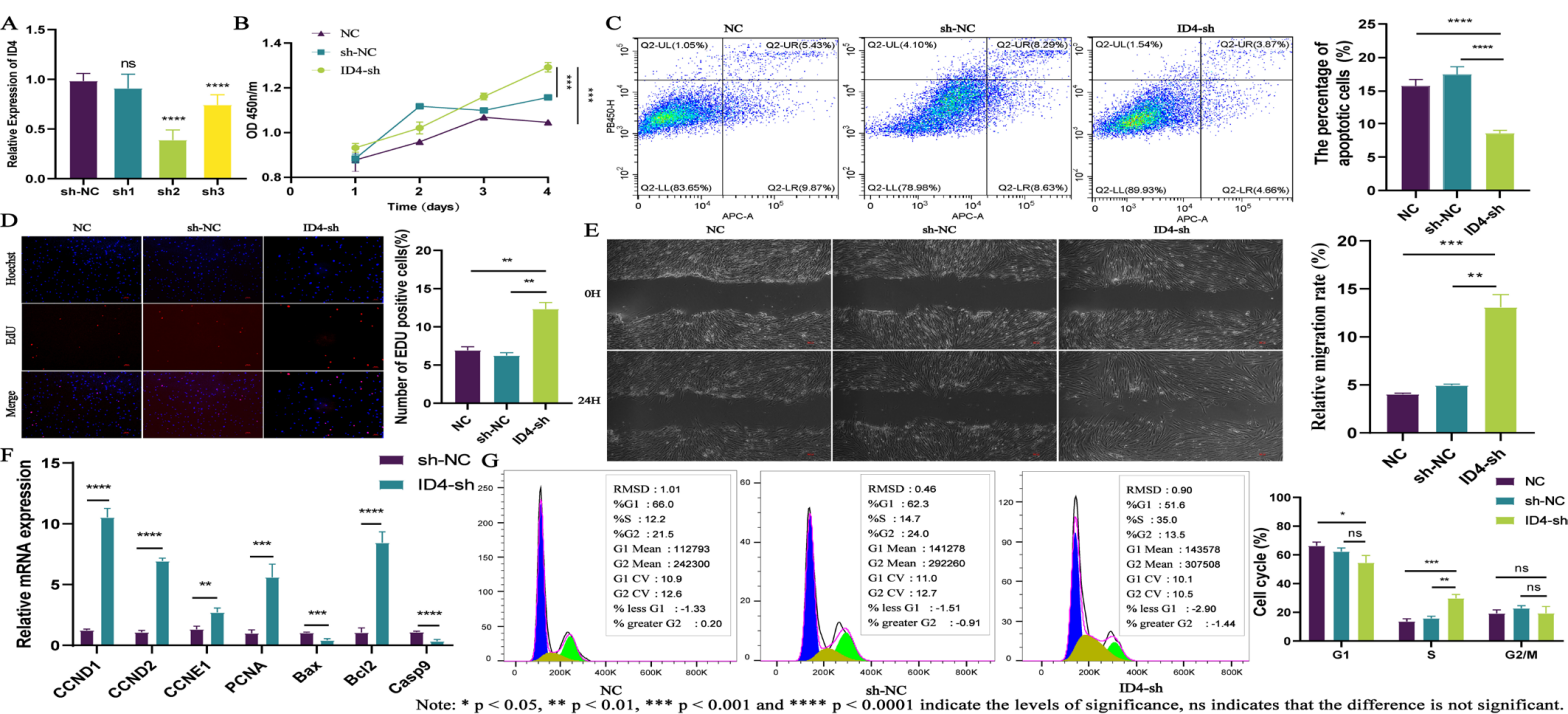
Functional analysis of *ID4* in dermal fibroblasts. (A) Screening of *ID4* interference vector. (B) CCK8 was used to detect the proliferation of the ID4-sh cell line. (C) The apoptosis of dermal fibroblasts was detected after *ID4* interference. (D) The EdU was used to detect the proliferation of the ID4-sh cell line. (E) The cell scratch assay was used to detect the migration of the ID4-sh cell line. (F) The expression of cell proliferation/apoptosis marker genes was detected by qRT-PCR. (G) DNA staining was used to detect the cell cycle of the lncRNA ID4-sh cell line. qRT-PCR, quantitative real-time reverse transcription polymerase chain reaction.

Subsequently, we constructed three *ID4* interference vectors and transfected them into dermal fibroblasts with lentiviral transfection. The experiment found that ID4-sh2 had the best interference efficiency, so we chose this cell line for the next experiments (Figure 4A). Firstly, we used methods such as CCK8, EdU, and Annexin V-APC/PI double staining to investigate the effects of *ID4* interference on the proliferation and apoptotic abilities of dermal fibroblasts. The experimental results showed that knocking down *ID4* can promote the proliferation of dermal fibroblasts and inhibit their apoptosis (Figures 4B–4D). These results were similar to those obtained following lncRNA ST6GALNAC3 knockdown, in line with the ceRNA hypothesis. Furthermore, experimental results from cell cycle analysis indicated that knocking down *ID4* increased the proportion of dermal fibroblasts in the S phase (Figure 4G). Additionally, qRT-PCR was employed to detect changes in the expression of marker genes related to cell proliferation and apoptosis in the cell lines. Our findings revealed that ID4-sh enhanced the expression of cell proliferation-related marker genes and anti-apoptosis marker genes, while simultaneously inhibiting the expression of apoptosis-promoting genes (Figure 4F). Experimental results from cell migration assays showed that the migration ability of dermal fibroblasts was significantly enhanced after transfection with ID4-sh (Figure 4E). Based on the aforementioned experimental results, knocking down *ID4* promoted the proliferation and migration abilities of dermal fibroblasts. This promotional effect was achieved by increasing the proportion of cells in the S phase and upregulating the expression of cell proliferation-related genes. In addition, we noted that the effects of ID4-sh and lncRNA ST6GALNAC3-sh on the phenotypes of dermal fibroblasts were consistent, which phenomenon is in line with the ceRNA hypothesis.

### Chi-miR-24-3p inhibitor can reverse the inhibitory effects of both lncRNA ST6GALNAC3 and *ID4* on the proliferation and migration of dermal fibroblasts

**Figure 5:**
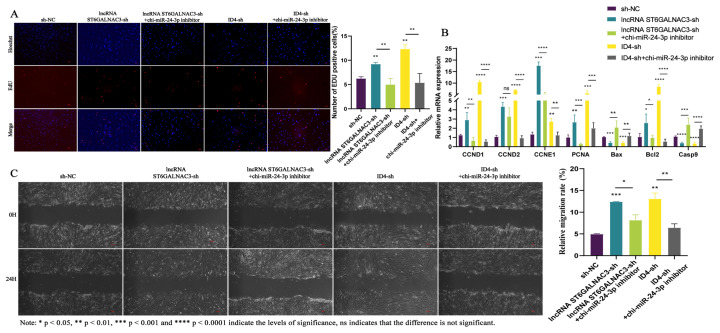
Chi-miR-24-3p can reverse the effect of lncRNA ST6GALNAC3-sh/ID4-sh on the cell phenotype of dermal fibroblasts. (A) The EdU assay was used to detect cell proliferation. (B) The expression of cell proliferation/apoptosis marker genes was detected by qRT-PCR. (C) The cell scratch assay was used to detect cell migration. qRT-PCR, quantitative real-time reverse transcription polymerase chain reaction.

**Figure 6:**
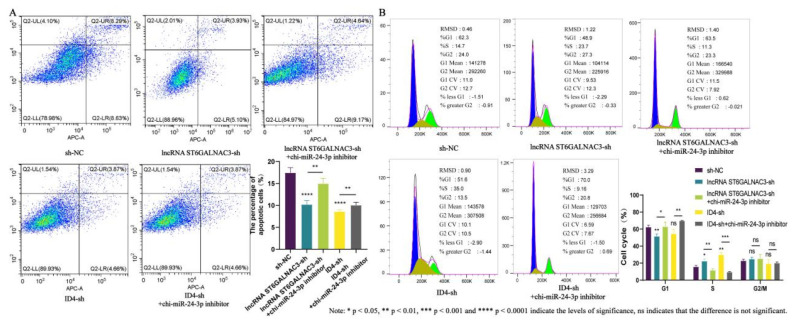
Chi-miR-24-3p can reverse the effect of lncRNA ST6GALNAC3-sh/ID4-sh on the cell phenotype of dermal fibroblasts. (A) Annexin V-APC/PI double staining was used to detect cell apoptosis. (B) DNA staining was used to detect the cell cycle.

Furthermore, to validate the lncRNA ST6GALNAC3-chi-miR-24-3p-ID4 regulatory network, we added chi-miR-24-3p inhibitor to both the lncRNA ST6GALNAC3-sh and ID4-sh cell lines and examined the resulting changes in cellular phenotypes. Previous experimental results demonstrated that both lncRNA ST6GALNAC3-sh and ID4-sh could enhance the proliferation and migration of dermal fibroblasts while inhibiting their apoptosis. After adding chi-miR-24-3p inhibitor to the lncRNA ST6GALNAC3-sh or ID4-sh cell lines, we observed a significant reduction in the proportion of EdU-positive cells in the co-transfected group compared to the singly transfected groups (Figure 5A). Simultaneously, the proportion of cells in the S phase and the expression levels of cell proliferation marker genes also significantly decreased. This indicates that the chi-miR-24-3p inhibitor can counteract the proliferation-promoting effects of lncRNA ST6GALNAC3-sh and ID4-sh on dermal fibroblasts (Figures 5B,6B). Next, we investigated cell apoptosis. The results of the apoptosis experiments demonstrated that, after adding chi-miR-24-3p inhibitor to the lncRNA ST6GALNAC3-sh and ID4-sh cell lines, there was a significant increase in the proportion of apoptotic cells, with the most pronounced increase observed in the proportion of early apoptotic cells (Figure 6A). The cell migration ability was evaluated through the cell scratch assay. As shown in Figure 5C, with the addition of chi-miR-24-3p inhibitor to the lncRNA ST6GALNAC3-sh and ID4-sh cell lines, the migration-promoting effect induced in these cell lines was subsequently counteracted.

**Figure 7:**
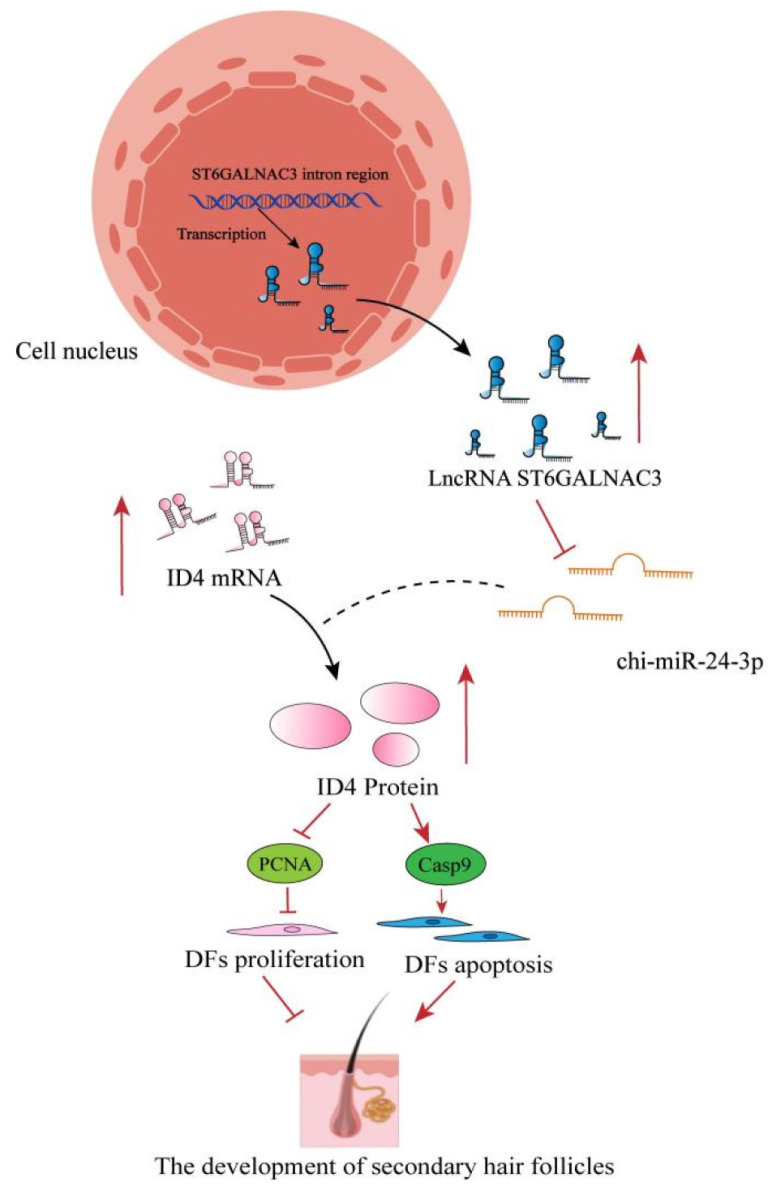
Diagram of the lncRNA ST6GALNAC3/chi-miR-24-3p/ID4 regulatory mechanism.

The comprehensive results indicate that chi-miR-24-3p, as a common target miRNA for both lncRNA ST6GALNAC3 and *ID4*, can rescue the proliferation and migration-promoting effects caused by lncRNA ST6GALNAC3-sh and ID4-sh. This suggests the existence of an lncRNA ST6GALNAC3-chi-miR-24-3p-ID4 regulatory axis in dermal fibroblasts. Additionally, in dermal fibroblasts, lncRNA ST6GALNAC3 can act as a sponge for chi-miR-24-3p, regulating the expression of *ID4*, and subsequently influencing the proliferation and migration abilities of dermal fibroblasts, thereby modulating the morphogenesis of secondary hair follicles (Figure 7).

## DISCUSSION

In the breeding industry of cashmere goats, cashmere constitutes a significant economic product, with its quality and yield being closely associated with hair follicle development. Hair follicles, as important appendages of mammalian skin, have complex structures and developmental characteristics that have always been a focus of biological research [3]. Dermal fibroblasts, as key cells in forming hair follicle structures, differentiate to form dermal papilla, which serve as the signaling hub for normal hair follicle development and directly determine the fate of hair follicles [19]. Therefore, exploring the phenotypic changes in dermal fibroblasts to gain a deeper understanding of the molecular mechanisms underlying hair follicle development has important practical significance for improving the quality and yield of cashmere.

Based on the previously constructed transcriptome database of skin samples from different embryonic stages of cashmere goats [13], this study screened out 158 lncRNAs related to secondary hair follicle morphogenesis and focused on lncRNA ST6GALNAC3. The study found that this lncRNA was significantly downregulated during secondary hair follicle morphogenesis and was mainly located in the cytoplasm of dermal fibroblasts. After interfering with lncRNA ST6GALNAC3, the proliferation and migration abilities of dermal fibroblasts were significantly enhanced, indicating that lncRNA ST6GALNAC3 has an important impact on the phenotypic characteristics of dermal fibroblasts. CeRNA is a novel mode of gene expression regulation. This hypothesis reveals a new mechanism of RNA-RNA interaction, which is that ceRNA molecules regulate mRNA expression by competitively binding to common miRNAs [20]. The ceRNA mechanism does not refer to a specific type of RNA molecule, but rather to a regulatory mechanism involving various types of RNA molecules, including mRNA, pseudogenes, lncRNAs, and circRNAs [20]. A substantial number of studies have found that during the development of hair follicles, lncRNAs often act as ceRNAs, influencing the proliferation and differentiation of hair follicle cells and thereby playing a crucial regulatory role [10]. In this study, through predictive analysis and experimental validation, we found that lncRNA ST6GALNAC3 can act as a sponge for chi-miR-24-3p, alleviating its inhibitory effect on the expression of *ID4* in dermal fibroblasts. This discovery uncovers the molecular regulatory mechanism of lncRNA ST6GALNAC3 in hair follicle development. Specifically, through its interaction with chi-miR-24-3p, lncRNA ST6GALNAC3 indirectly modulates the expression of *ID4*, which subsequently affects the biological functions of dermal fibroblasts, ultimately influencing hair follicle formation.

*ID4*, an important member of the TGF-β signaling pathway, plays a pivotal role in hair follicle morphogenesis during the embryonic period of cashmere goats. This study further elucidates the regulatory network of lncRNA ST6GALNAC3/chi-miR-24-3p/ID4 at the cellular level. Silencing either *ID4* or lncRNA ST6GALNAC3 significantly enhanced the proliferation and migration of dermal fibroblasts, indicating that this regulatory network plays a crucial role in regulating the biological functions of dermal fibroblasts during hair follicle development. However, we performed a series of rescue experiments after adding chi-miR-24-3p inhibitor to lncRNA ST6GALNAC3-sh or ID4-sh cell lines. The results showed that the promoting effects of lncRNA ST6GALNAC3-sh or ID4-sh on the proliferation and migration of dermal fibroblasts were significantly reversed, indicating that lncRNA ST6GALNAC3 indirectly regulates the expression of *ID4* through chi miR-24-3p, thereby affecting hair follicle development. Compared with previous related studies, this study not only clarifies the role of lncRNA ST6GALNAC3 in the hair follicle development of cashmere goats but also elaborates on its underlying molecular regulatory mechanism, thereby providing more specific research ideas and methods for further investigating the functions of lncRNAs in hair follicle development. However, this study has certain limitations. Firstly, the functions and regulatory mechanisms of lncRNA ST6GALNAC3 were only studied at the cellular level and have not been validated *in vivo*. Future studies can further verify the role of lncRNA ST6GALNAC3 in hair follicle development by constructing animal models. Secondly, although this study reveals the regulatory network of lncRNA ST6GALNAC3/chi-miR-24-3p/ID4, other regulatory factors and signaling pathways involved in hair follicle development may require further investigation.

In summary, this study reveals the important role and molecular regulatory mechanism of lncRNA ST6GALNAC3 in cashmere goat hair follicle development, providing a novel theoretical basis for exploring the molecular mechanisms of cashmere goat hair follicle development. Future research can build on this foundation to further explore the role of lncRNAs in hair follicle development, providing more powerful technical support for improving the quality and yield of cashmere.

## CONCLUSION

This study systematically analyzed the regulatory role of lncRNA ST6GALNAC3 in the morphogenesis of secondary hair follicles in cashmere goats. The experimental results have been verified to demonstrate that lncRNA ST6GALNAC3 can indirectly regulate the expression of *ID4* via its interaction with chi-miR-24-3p, thereby inhibiting the proliferation and migration of dermal fibroblasts and subsequently impeding the morphogenesis of secondary hair follicles (Figure 7).
